# Grade V Liver Injury Presented With Peritonitis Treated With Stapler-Assisted Hepatic Segmentectomy: A Case Report

**DOI:** 10.7759/cureus.41436

**Published:** 2023-07-06

**Authors:** Laura Mena Albors, Samantha Reiss, Adam Shen, Darwin Ang

**Affiliations:** 1 General Surgery, University of Central Florida College of Medicine, Orlando, USA; 2 Medical School, University of Central Florida College of Medicine, Orlando, USA; 3 Surgery, Hospital Corporation of America (HCA) Florida Ocala Hospital, Ocala, USA; 4 Trauma, Hospital Corporation of America (HCA) Florida Ocala Hospital, Ocala, USA; 5 Trauma, University of South Florida, Tampa, USA

**Keywords:** liver trauma, liver rupture, operative skills, blunt abdominal, blunt liver trauma

## Abstract

The liver is one of the most commonly injured solid organs in blunt abdominal trauma. In patients who are hemodynamically normal, most cases of blunt liver injuries are managed conservatively. At present, nonoperative management (NOM) is the standard of care for both minor and severe liver injuries. Usually, patients with severe liver injuries, i.e., grades IV and V, are treated with surgical intervention versus angioembolization depending if patients are hemodynamically stable or not. We present a hemodynamically stable 53-year-old male patient with a grade V blunt liver injury with complete avulsion of the left lobe of the liver after a motor vehicle collision (MVC). Very few cases of complete hepatic avulsions have been published in the literature. We discuss surgical management with stapler-assisted hepatectomy in emergency trauma laparotomy for bleeding control.

## Introduction

The liver is one of the most commonly injured solid organs in blunt abdominal trauma due to its large size and anterior positioning inferior to the subcostal margin. Nonoperative management (NOM) is the standard of care for both minor and severe liver injuries [1-2]. Automobile accidents account for a significant portion of blunt injuries to the liver [3]. Liver injuries can be classified using the liver injury scale of the American Association for the Surgery of Trauma (see Appendix) [4]. Grades I-III are typically considered mild injuries, whereas grades IV and V are considered severe. Usually, mild injuries are managed conservatively. The management of severe injuries is more controversial due to their higher mortality rate and increased risk of complications [5].

The treatments of blunt liver injuries can range from NOM to surgery. Angioembolization (AE) by interventional radiology (IR) typically functions as a bridge between the two [6]. Currently, 50% to 85% of blunt liver injuries can be treated nonoperatively with or without IR. All grades of liver injuries can be treated conservatively as long as the patient is stable; however, patients with severe liver injuries often have trauma-related complications that impact their hemodynamic stability [7].

Compared to mild liver injuries, severe liver injuries more commonly require surgery [8]. These grades of liver injuries often present with multiple injuries and shock [9]. Their reported mortality rate is above 40% [10] . Bleeding from a non-hepatic source, damage to additional abdominal organs, and significant destruction or avulsion of portions of the liver are conditions where surgery is necessitated [11]. Very few cases of complete hepatic segment avulsions have been published in the literature [6]. This case report details the diagnosis and management of a patient with severe avulsion to the left lobe of the liver.

## Case presentation

A 53-year-old male with a past medical history of chronic obstructive pulmonary disease (COPD) presented as a trauma alert after a high-velocity motor vehicle collision (MVC) in which he was an unrestrained driver. He self-extricated and was found lying outside of the car. As a result of the MVC, he sustained a blunt impact to the chest and abdomen from the steering wheel. The patient was normotensive upon arrival and had a Glasgow Coma Score (GCS) of 14. He was complaining of severe abdominal pain with peritonitic findings on exam. The patient underwent a focused assessment with sonography for trauma (FAST) exam. The FAST was positive in the right and left upper quadrants. 

**Figure 1:**
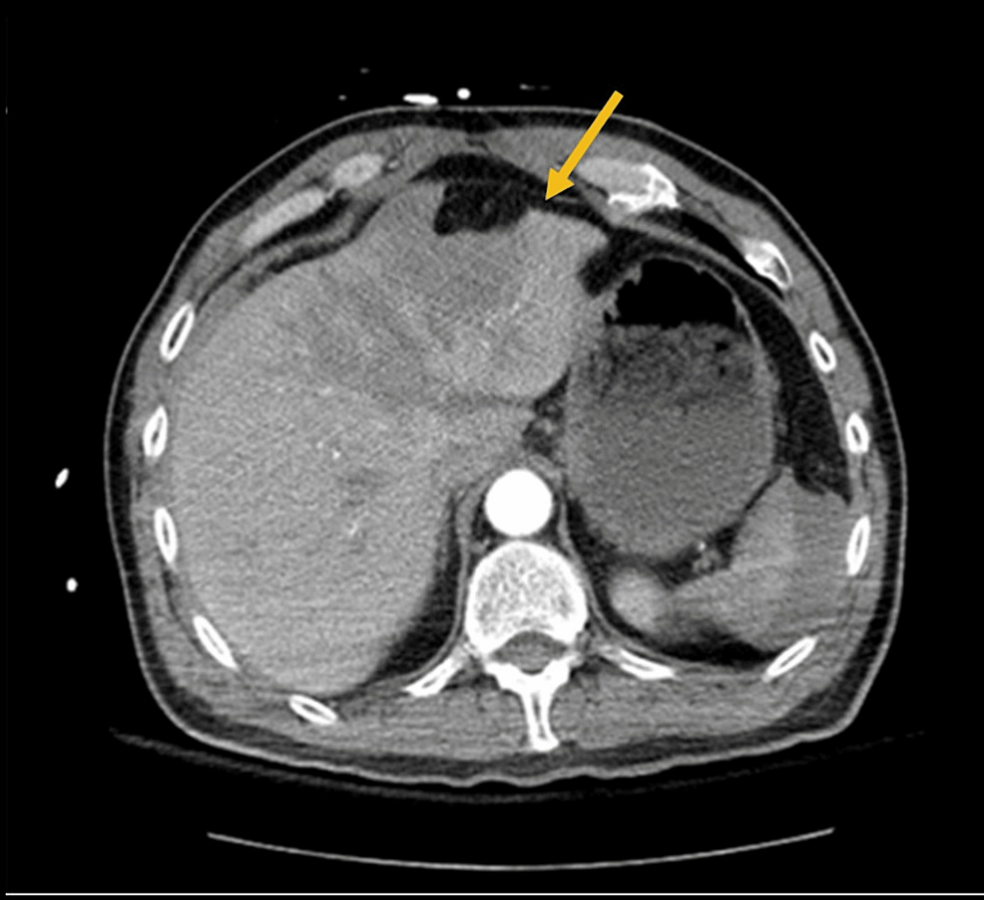
Grade V liver laceration, more than 75% of liver parenchymal disruption of the hepatic lobe. Parenchymal disruption showed by the arrow.

**Figure 2:**
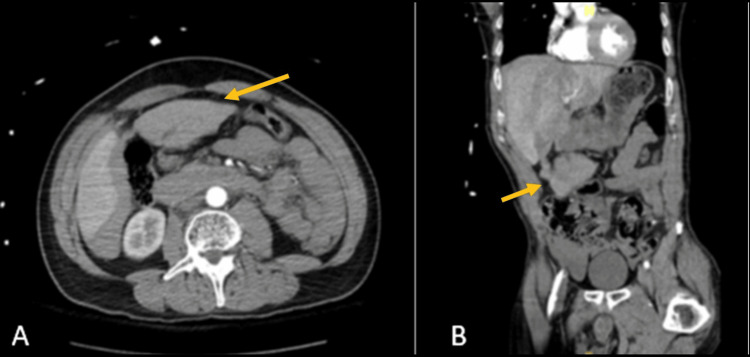
Grade 5 liver laceration with a complete avulsion of the left lobe and hemoperitoneum (A: axial view; B: coronal view). The arrow shows the complete avulsion of the separated left lobe.

**Figure 3:**
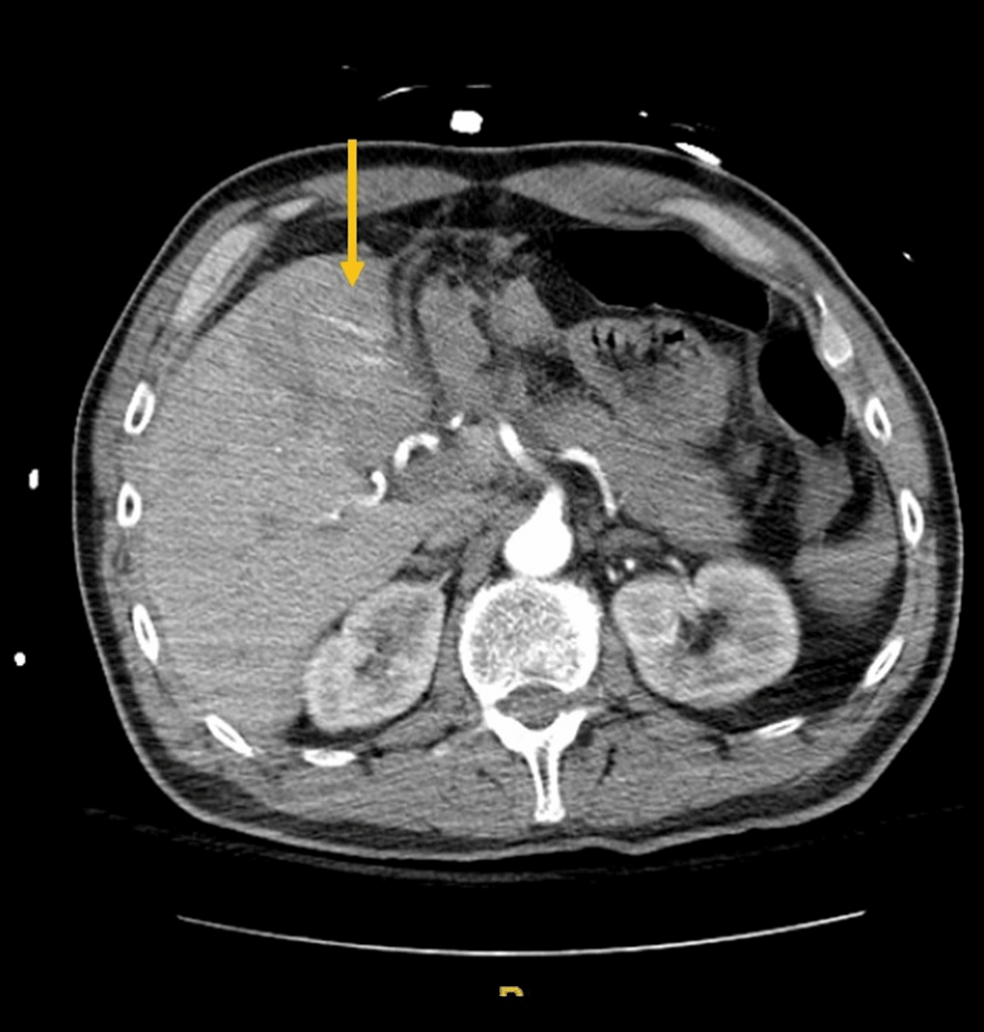
Active bleeding beyond the confines of the liver (arrow).

As the patient remained stable, he underwent a computed tomography (CT) scan for further evaluation. The CT scan showed a grade V liver laceration involving more than 75% of the left lobe of the liver (Figure 1), with a significant section of the inferior left lobe, measuring 8 cm x 5 cm x 7 cm, completely detached from the remaining portion of the liver and displaced inferiorly by several centimeters (Figure 2). The CT scan also showed evidence of active bleeding beyond the confines of the liver with a significant hemoperitoneum (Figure 3). The patient was immediately taken to the operating room for the evaluation of a hollow viscus organ injury in the presence of peritonitis and for definitive hemorrhage control.

In the operating room, a midline laparotomy was performed. The falciform ligament was immediately divided. A significant portion of the left lobe of the liver was noted to be completely detached from the remaining liver. A superficial serosal tear at the pylorus and a non-expanding mesenteric hematoma of the transverse colon were the second traumatic findings noted. The abdomen was packed in the usual trauma protocol. Pulsatile bleeding was identified coming from gastroduodenal artery (GDA), which was ligated. The remainder of the left lobe of the liver was assessed and determined to be non-salvageable with significant parenchymal bleeding, as it was mostly avulsed from the umbilical fissure. The Pringle maneuver and manual compression were performed at the edge of the right lobe of the liver. The parenchymal bleeding was unable to be controlled with electrocautery, so a decision was made to perform a left lateral segmentectomy. This was achieved using an endoscopic 60 mm gastrointestinal anastomosis stapler with green load on the liver parenchyma and left hepatic pedicle after lowering the hilar plate. The rest of hemostasis after stapling was obtained by electrocautery, silk suture, and a fibrin-based hemostatic agent. No further injury was found when the rest of the abdomen was explored. Two closed-suctioned surgical drains were placed above and below the liver. No bile leaks were observed, and the abdomen was closed.

Intraoperatively, the patient received 3200 mL IV fluid, two packed red blood cells, two fresh frozen plasma, and 223 mL blood from a cell saver. No intraoperative vasopressors were needed as the patient continued to be hemodynamically stable in the intraoperative period. He was extubated postoperative day (POD) 1. On POD 2, a hepatobiliary iminodiacetic acid (HIDA) scan was performed before advancing diet, and no bile leak was noted in the study. Diet was slowly advanced, and on POD 6, the patient developed increased abdominal pain, nausea, and vomiting concerning for the development of the bile leak. On physical exam, 280 mL of bile output was noted through the close-suction surgical drains. The patient underwent an endoscopic retrograde cholangiopancreatography (ERCP) on POD 7 for stent placement in the common bile duct. On POD 12, the patient was tolerating a diet and ambulating. The bile drainage from the drains was minimal. The patient was discharged to home and instructed to follow up with a surgeon in his local area, as he was from a different state.

## Discussion

The presentation of patients with high-grade liver injuries is variable, and the management of such injuries is controversial [5]. Patients who are hemodynamically normal, like the patient presented here, are often directed toward NOM or AE. Even though our patient presented hemodynamically stable, he also presented with a few key factors that necessitated surgical intervention instead of NOM: concerns for a hollow viscus injury for peritonitis on the exam and complete detachment of the liver segment. 

Surgical options for severe liver trauma include hepatectomy, nonanatomic resection, and anatomic resection [6]. Although a few studies have had success with liver resection, many surgeons opt for a more conservative approach during surgery due to a reported mortality rate of 50% with resection [12]. Nonanatomic resection can further be classified as partial resection or resectional debridement. Resectional debridement removes only nonviable tissues at the site of injury, whereas partial resection completes the resection started by the trauma to form a smooth surface in addition to removing the nonviable tissues [13]. Nonanatomic resection is typically selected over anatomic resection in trauma surgery because it is safer and less challenging [14]. Anatomic resection involves the removal of one or more of Couinaud’s liver segments instead of following the line caused by the liver trauma. According to Strong et al., anatomic resection is useful for removing any sites of hepatic bleeding and areas of necrosis, leaving only viable hepatic tissues behind, which reduces the chance for septic complications [15].

Both nonanatomic and anatomic resection can be completed with staplers [14]. Stapling devices can be advantageous in trauma situations. They can reduce the operative time and control bleeding and bile leaks. In elective hepatectomies, such as segmentectomies and wedge resections, resection using a GIA stapler with vascular loads has been shown to be successful [16]. However, stapler usage for hepatic resection in trauma cases has rarely been reported in the literature. In those reported, GIA staplers with green loads had been successfully used for nonanatomic resections [17]. The resection of hepatic segments results in loss of the functioning liver tissue. Following hepatic resection, the liver will usually regenerate the mass of the removed lobes within two weeks, restoring the liver functionality but not the anatomic structure [2,18].

Our patient required an ERCP with stent placement on POD 7 due to the bile output in the drains. Although bile leaks have been reported to be lower with GIA stapler usage for hepatic resection due to the compressive sealing of the bile ducts, bile leakage is still a common complication of hepatic resection both in stapler hepatectomies and in standard hepatectomies. The patient underwent an ERCP with stent placement. ERCP with stent placement has been reported to be successful in treating patients with biliary complications following hepatic surgery [2,14].

## Conclusions

Grade V liver laceration injuries are difficult to manage and have a high mortality rate. Very few cases of complete hepatic segment avulsions have been published. This case report demonstrates a rare case of grade V liver laceration with a complete avulsion of a liver segment treated with nonanatomic resection using a GIA stapler with good outcomes. It highlights the safe use of GIA staplers with a green load in the setting of trauma when a vascular load is not available.

## Appendices

**Table 1:** Liver injury scale of the American Association for the Surgery of Trauma (2018) Source: Kozar RA, Crandall M, Shanmuganathan K, et al.: Organ injury scaling 2018 update: spleen, liver, and kidney. J Trauma Acute Care Surg. 2018, 85:1119-22.

Grade	Criteria
I	Subcapsular hematoma <10% surface area
	Parenchymal laceration <1 cm depth
	Capsular tear
II	Subcapsular hematoma 10-50% surface area; intraparenchymal hematoma <10 cm in diameter
	Laceration 1-3 cm in depth and ≤10 cm length
III	Subcapsular hematoma >50% surface area or expanding; ruptured subcapsular or parenchymal hematoma
	Intraparenchymal laceration >10 cm
	Laceration >3 cm depth
	Any injury in the presence of a liver vascular injury or active bleeding contained within liver parenchyma
IV	Parenchymal disruption involving 25-75% of a hepatic lobe
	Active bleeding extending beyond the liver parenchyma into the peritoneum
V	Parenchymal disruption >75% of hepatic lobe
	Juxtahepatic venous injury to include retrohepatic vena cava and central major hepatic veins
